# Philadelphia Hospitals

**Published:** 1844-05

**Authors:** 


### THE WESTERN JOURNAL.

New Series.—Vol. I.—No. V.

LOUISVILLE, MAY 1, 1844.

### PHILADELPHIA HOSPITALS.

Dr. Bell, the judicious and able editor of the Bulletin of Medical Science, represents the teaching of Clinical Medicine in the Philadelphia Hospitals as having been meagre and imperfect during the last winter. The amphitheatre exhibitions made by the Professors in the two schools he considers poor substitutes for bed-side medicine, or clinics proper. It seems that the wards of the Pennsylvania Hospital were almost deserted by the students, and that but one visit a week was made to the Philadelphia Hospital. These signs, he thinks, indicate anything but a healthy state of clinical instruction. He adds:

"If defects in the regular system hitherto pursued have been discovered, let them be amended by consultation between Professors of Medical Schools and Managers of Hospitals and Dispensaries; but in the name of consistency, let not the philosophy and true nobility of medicine be tarnished by empirical devices, expedients of the hour, to the sacrifice of her future character and usefulness. In common with every Philadelphian, and especially every Philadelphia physician, we find ourselves compromised by the present course and tendency of clinical exhibitions once a week within the walls of a college."

Dr. Bell has here stated a grievance more easily pointed out than remedied. How clinical medicine may be taught satisfactorily to a large class, we take to be one of the unsolved problems which lie in the way of medical teachers. The old method of congregating several hundred young men in the wards of a hospital, where, at most, only a dozen or two could either see the patient or hear the words of the professor, was too ridiculous to be long continued. The amphitheatre has now, we believe, been pretty generally adopted, and is esteemed a great improvement upon the former system. Still it is liable to many serious objections. It is desirable that each student should make a personal examination of the patient, which, in fact, is the only course by which he can become instructed in the signs and diagnosis of disease. He who shall indicate a plan by which this object can be carried into successful operation, will be a benefactor to the medical profession. Clinical medicine is justly regarded as an indispensable branch of medical instruction, and it is the interest of every medical school to foster and develope it. A school in which it is not taught would now be esteemed radically defective in its organization—could hardly be called respectable. We are glad to perceive a growing disposition among students to pass the summer in the neighborhood of hospitals, and avail themselves of clinical instruction during the months when the wards offer the greatest variety of interesting disease, and the best opportunities for deliberate individual inspection. The class in attendance on the summer lectures, in this city, is encouraging, and pupils are coming in every few days. The Louisville Marine Hospital affords excellent facilities for clinical instruction, of which it is hoped that the gentlemen engaged in teaching it will make profitable use.

